# The Chinese ACL injury population has a higher proportion of small ACL tibial insertion sizes than Western patients

**DOI:** 10.1007/s00167-019-05541-z

**Published:** 2019-05-20

**Authors:** Feilong Li, Leilei Qin, Xuan Gong, Zhenggu Huang, Ting Wang, Ziming Liu, Steve Sandiford, Jianye Yang, Sizheng Zhu, Xi Liang, Wei Huang, Ning Hu

**Affiliations:** 1 Department of Orthopedics, The Dazu District People’S Hospital, Chongqing, 402360 China; 2 Department of Orthopedics, The First Affiliated Hospital of Chongqing Medical University, Chongqing, 400016 China; 3 Department of Outpatient, Chongqing General Hospital, Chongqing, 400013 China; 4 Department of Orthopedics, Queen Elizabeth Hospital, Bridgetown, Barbados

**Keywords:** Anterior cruciate ligament (ACL), Intercondylar notch width, Insertion size

## Abstract

**Purpose:**

The study purpose is to characterize the sizes of the anterior cruciate ligament (ACL) insertion site and intercondylar notch in Chinese patients undergoing ACL surgery. The findings will provide a reference for individualized clinical treatment of ACL rupture.

**Methods:**

For this study, 137 patients (102 males, 35 females) with an average age of 30.3 ± 9.5 years (range 14–52 years) undergoing ACL reconstruction were included. The tibial ACL insertion site length and width and the intercondylar notch width were measured on MRI and arthroscopically using a ruler. Descriptive statistics of the patients, the distribution of the measurements and the differences between males and females were calculated.

**Results:**

The ACL tibial insertion size and intercondylar notch width in Chinese patients with ACL injuries, as obtained by MRI and intra-operatively, exhibited significant individual variability. The tibial ACL insertion site had a mean length of 13.5 ± 2.1 mm and width of 10.9 ± 1.5 mm as measured on MRI and a mean length of 13.3 ± 2.1 mm and width of 11.0 ± 1.6 mm as measured intra-operatively. The mean intercondylar notch width was 15.2 ± 2.4 mm on MRI and the mean length was 15.0 ± 2.5 mm intra-operatively. The inter-rater reliability between MRI and intra-operative measurements confirmed that the two methods were consistent. In 65.7% of individuals, the ACL tibial insertion length was < 14 mm.

**Conclusion:**

The distribution of tibial footprint size in Chinese patients is different from that in Western populations. There is a higher proportion of subjects with a tibial footprint size < 14 mm among Chinese patients with ACL injury. Therefore, great care should be taken when treating this population with the double-bundle technique or larger graft options.

**Level of evidence** IV.

## Introduction

Due to its unique anatomical structure and effect, rupture of the anterior cruciate ligament (ACL) is relatively common, with the mainstay of treatment being ACL reconstruction (ACL-R). In recent years, anatomical and biomechanical considerations have resulted in the preference of surgery to restore the anatomy of the ACL [7, 41]. Many anatomical and biomechanical studies have defined the characteristics of the native state of the ACL, which is housed in the femoral intercondylar notch [48]. Functionally, it has two distinct bundles [49], the anteromedial (AM) bundle and the posterolateral (PL) bundle. However, some instances showed that the ACL insertion site or intercondylar notch size may be too small to accommodate both grafts in a double-bundle (DB) reconstruction [31, 36, 41]. These factors have influenced the selection of grafts and techniques, resulting in a more individualized approach to ACL-R [22, 43].

Specifically, some researchers have suggested that in patients with a tibial insertion site < 14 mm in length and a notch < 12 mm in width, the native anatomy may be better reproduced with single-bundle (SB) ACL-R [15, 28–30, 44]. The proportion of individuals with an ACL tibial footprint size < 14 mm in length was reportedly low, varying between 3.6% and 10.6% in Western patient populations [22, 39, 47]. However, recent data revealed that the distribution of the size of the ACL tibial footprint widely varied among different ethnic groups; for example, there is a high proportion (53.5%) of South Korean females with a tibial footprint length < 14 mm [27]. We assert that ACL anatomical differences may also occur in Chinese patients, as the ACL tibial footprint in the Chinese population has yet to be thoroughly investigated [27, 34].

Some attempts have been made to address those considerations in similar populations, while there are still limitations, such as relatively small sample sizes, cadaveric specimens of elderly persons, and osteoarthritic patients undergoing total knee arthroplasty [17, 23, 26, 27]. Moreover, considering the previous study showing that patients who have suffered a non-contact ACL injury had a significantly smaller ACL volume in their contralateral knee than the volume of gender-, height-, age-, and weight-matched normal controls [6], and it is possible that a link exists between ACL size and injury risk [45]. Anatomical ACL data may need to be re-evaluated in the Chinese population of patients with ACL injury. Therefore, the following hypotheses are proposed:

1. The ACL insertion site and intercondylar notch size have significant size differences compared to those in Western populations.
2. A higher proportion of ACL insertions is < 14 mm in Chinese patients with ACL rupture than in Western patients.

The purpose of this study was to identify the ACL insertion site and intercondylar notch size in Chinese patients undergoing ACL surgery. The data will provide a reference for individualized ACL graft production, technical modifications for treatment of Chinese patients with ACL rupture and a clear determination for choosing between SB ACL-R or DB ACL-R in the Chinese population of patients with ACL injuries.

## Materials and methods

The study was carried out from November 2017 to July 2018. All methods were performed in accordance with the ethical guidelines. Informed consent was obtained from all participants.

The main inclusion criteria were recent patients with ACL rupture and reconstruction within 3 months after the injury. The exclusion criteria were as follows: patients with high-energy injuries, such as car accidents and falls, patients with multiple ligamental injuries, patients with severe osteoarthritis (grade III or higher), patients with tuberculosis of the knee, suppurative arthritis, rheumatoid arthritis, or with tumour, or those who underwent previous notchplasty [17, 26, 47, 48]. Patients with concomitant injuries such as a meniscus tear or an MCL rupture were not excluded because the notch width and insertion site size of the ACL were not influenced.

One hundred and thirty-seven patients (102 males, 35 females) with an average age of 30.3 ± 9.5 years (range 14–52 years) were included in this study. All patients underwent preoperative MRI and ACL-R at the First Affiliated Hospital of Chongqing Medical University.

### Preoperative MRI measurement

Patients were scanned using a 1.5-T magnet (Signa HDxt, GE Medical System, USA). Images were acquired in 4-mm slices using a flexible phased-array coil and clinically established MRI sequences at the Radiology Department. These views were obtained with the patient in the supine position and the knee in extension. T1-weighted fast spin-echo (FSE) images were obtained in all patients (TE, min full; TR, 760 ms; matrix size, 512*512; bandwidth, 31.25 kHz; field of view, 480 mm; slice thickness, 4 mm; spacing, 0.5 mm). T2-weighted fast recovery with fast spin-echo (FRFSE) sequence images were also obtained in each patient (auto TR, 2820 ms; echo train length (18 mm); matrix size, 512*512; bandwidth, 31.25 kHz; field of view, 480 mm; frequency, 228 kHz; slice thickness, 0.5 mm; spacing, 1 mm). In the coronal and sagittal planes, the images were oriented with the same anatomical alignment as the ACL and the femoral notch were, which allowed for a clear and predictable recognition of partial ACL tears and femoral notch shapes.

**Fig. 1:**
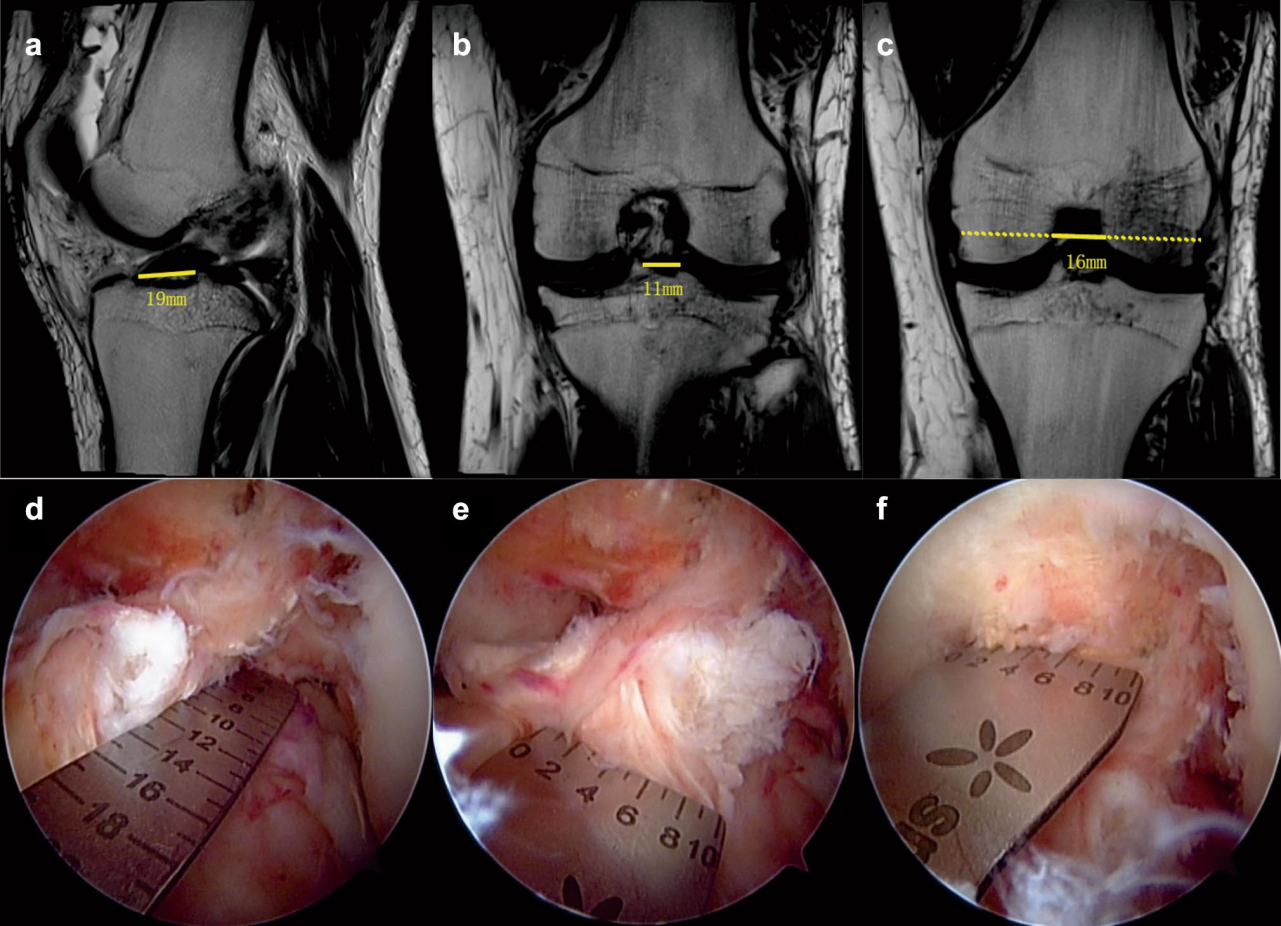
The nether pictures are arthroscopic views of a knee in flexion. The length (**d**) and width (**e**) of the notch (**f**) are measured using an arthroscopic ruler. A T2 proton density-weighted sequence of knee MRI showing a rupture of the ACL and the knee intercondylar notch. **a** The yellow line signifies the tibial ACL insertion site length. **b** The yellow line signifies the tibial ACL insertion site width. **c** The full yellow line signifies the knee intercondylar notch width

Currently, MRI is used not only for diagnosis but also for the measurement of the size and inclination angle of the ACL, as well as intercondylar notch size [1, 26, 46]. These data were compared with the intra-operative measurements performed by the surgeon using a specialized ruler and demonstrated high intra-rater reliability [46]. Single coronal and single sagittal proton density images were selected. The image with the most suitable sequence for ACL insertion size measurement was determined as the image that illustrated the ruptured ACL fibre attachment to the tibia [1]. The distance between the most anterior and most posterior fibres of the ACL attachment was determined to measure the exact length of the tibial ACL insertion site (Fig. 1a). The distance between the most medial and the most lateral portion of the ACL attachment was measured as the exact width of the tibial ACL insertion site (Fig. 1b). The coronal image of the most suitable sequence for intercondylar notch width measurement was the initial cut, which showed a clear and continuous image of the medial and lateral condyles (Fig. 1c) [26]. The initial image had a clear notch shape and a clear popliteus tendon sulcus of the lateral condyle. A line on the popliteus tendon sulcus was drawn. This line paralleled the lowest points of the cartilage surface of the medial and lateral condyles. The distance between the line and the crossing point of the medial and lateral condyles was measured as the intercondylar notch width [26]. A blinded observer in the Department of Orthopaedic Surgery (L.F.L.) performed the radiographic evaluations. The measurement was performed using a straight line and was rounded to the nearest 1 mm (Fig. 10). These results were measured three times, and the average value was used and also rounded to the nearest 1 mm.

### Intra-operative measurement

The intra-operative measurements were made by the senior surgeon. The three accessory medial, anterolateral, and anteromedial arthroscopic portals were established, which allowed nearly parallel-course positioning of the arthroscopic ruler with the tibial ACL remnant fibres and notch. A previous study demonstrated that the three-portal approach could achieve optimal arthroscopic visualization of the ACL insertion sites and intercondylar notch and had excellent accuracy for measuring the sizes of the ACL insertion sites and the intercondylar notch [15, 28, 46, 48]. The surgeon started with routine diagnostic arthroscopy of the knee and initial resection of the disruptive fat pad. The notch and the ACL stumps were clearly visualized [2]. To measure the ACL tibial insertion site, meticulous dissection of the distal remnant tibial ACL fibres was performed with care to preserve the borders of the insertion sites dissected to obtain the best view. Measurements were obtained of the native ACL tibial insertion site length, mid-width with a flexible, reusable, arthroscopic ruler (Smith and Nephew Endoscopy, Andover, Massachusetts), which was graded in millimetres (Fig. 1d, e). Measurements of the intercondylar notch, specifically the width at the middle of the notch, were then obtained using a ruler [29] (Fig. 1f). The senior surgeon (N.H.) performed all the operations and finished intra-operative measurements together with the other surgeons.

This clinical trial was approved by the Institutional Review Board and Hospital Ethics Committee of the First Affiliated Hospital of Chongqing Medical University (2017–191).

### Statistical analysis

An a priori sample size calculation was performed for the primary aim. The variables used for the calculation were based on findings of previously reported studies on notch and insertion site sizes (intercondylar notch width: 15.7 ± 2.6 mm; length of the tibial ACL insertion site: 15.36 ± 2.33 mm; width of the tibial ACL insertion site: 11.03 ± 1.77; significance: 0.05; power: 0.9) [23, 26]. This analysis showed that a minimum of 70 subjects should be included in the study. Descriptive statistics were calculated for the ACL tibial insertion site length and width, the notch width at the middle, and the patient age, height, and weight. The mean values, standard deviations, and ranges of measurements were evaluated. The frequency distributions, especially for the ACL tibial insertion size and notch width, were calculated. Differences in males and females were determined through an unpaired *t* test. Additional Bland–Altman plots were utilized to detect the agreement between the MRI and intra-operative measurements. All statistical analyses were performed with IBM SPSS version 23.0 software (SPSS Inc., an IBM Company, Chicago, Illinois, USA). The level of significance was determined at *P* < 0.05.

## Results

Age and anthropometric data, including height and weight of the included study subjects (*n* = 137), are given in Table 1. The mean age of the 137 patients evaluated was 30.3 ± 9.5 years (14–52 years), with a mean height and mean weight of 170.1 ± 8.3 cm (138–187 cm) and 71.3 ± 13.8 kg (40–110 kg), respectively. A total of 102 patients were men, and 35 were women.

**Table 1:** Demographic data

	Mean	SD	Minimum	Maximum
Age (years)	30.3	9.5	14	52
Height (cm)	170.1	8.3	138	187
Weight (kg)	71.3	13.8	40	110
BMI (kg/m^2^)	24.6	4.1	16.2	45.4
Males (%)	74.5			

MRI measurements of ACL length and width at the tibial attachment and of the intercondylar notch width, as well as the intra-operative measurements using an arthroscopic ruler are given in Table 2. The average ACL length and width at the tibial attachment on MRI were 13.5 ± 2.1 mm and 10.9 ± 1.5 mm, respectively. The average intercondylar notch width was 15.2 ± 2.4 mm. The anthropometric measurements are also shown in Table 2.

**Table 2:** Descriptive statistics (*n* = 137)

	MRI				Arthroscopic			
	Minimum	Maximum	Mean	SD	Minimum	Maximum	Mean	SD
Tibial insertion length	9	20	13.5	2.1	9	19	13.3	2.1
Tibial insertion width	8	15	10.9	1.5	8	15	11.0	1.6
Notch width	9	21	15.2	2.4	8	20	15.0	2.5

**Fig. 2:**
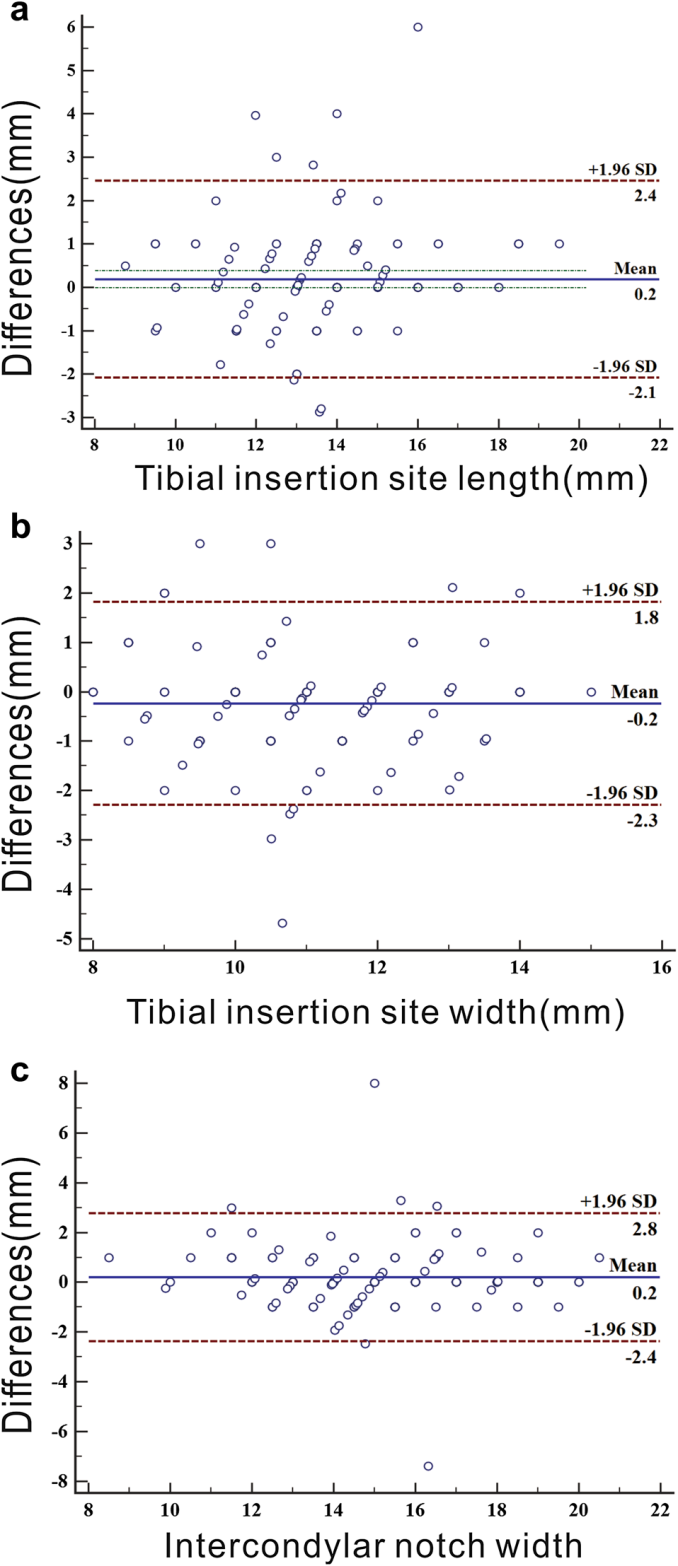
Bland–Altman plot: agreement between the MRI and intra-operative measurements of tibial insertion site length, tibial insertion site width and intercondylar notch width. Values of 5.8% (**a**, length of ACL insertion site), 6.5% (**b**, width of ACL insertion site), and 4.3% (**c**, width of intercondylar notch) are outside the limits of agreement

As expected, the Bland–Altman plot between the MRI and intra-operative measurements also showed excellent agreement (Fig. 2).

Table 3 shows the comparison of the gender subgroups regarding the notch width and ACL insertion site width and length. In terms of gender-related differences, the average male intercondylar notch width was significantly larger (15.5 ± 2.4 mm) than that of the female knees (14.5 ± 2.2 mm) (*P* < 0.05), and there were no significant gender differences in ACL insertion size.

**Table 3:** Differences in the notch and insertion site characteristics between male and female subjects

	Females (*n* = 35)	Males (*n* = 102)	*p* value
	Mean	Mean	
Tibial insertion length	12.9	13.8	n.s
Tibial insertion width	10.7	10.9	n.s
Notch width	14.5	15.5	< 0.05

**Fig. 3:**
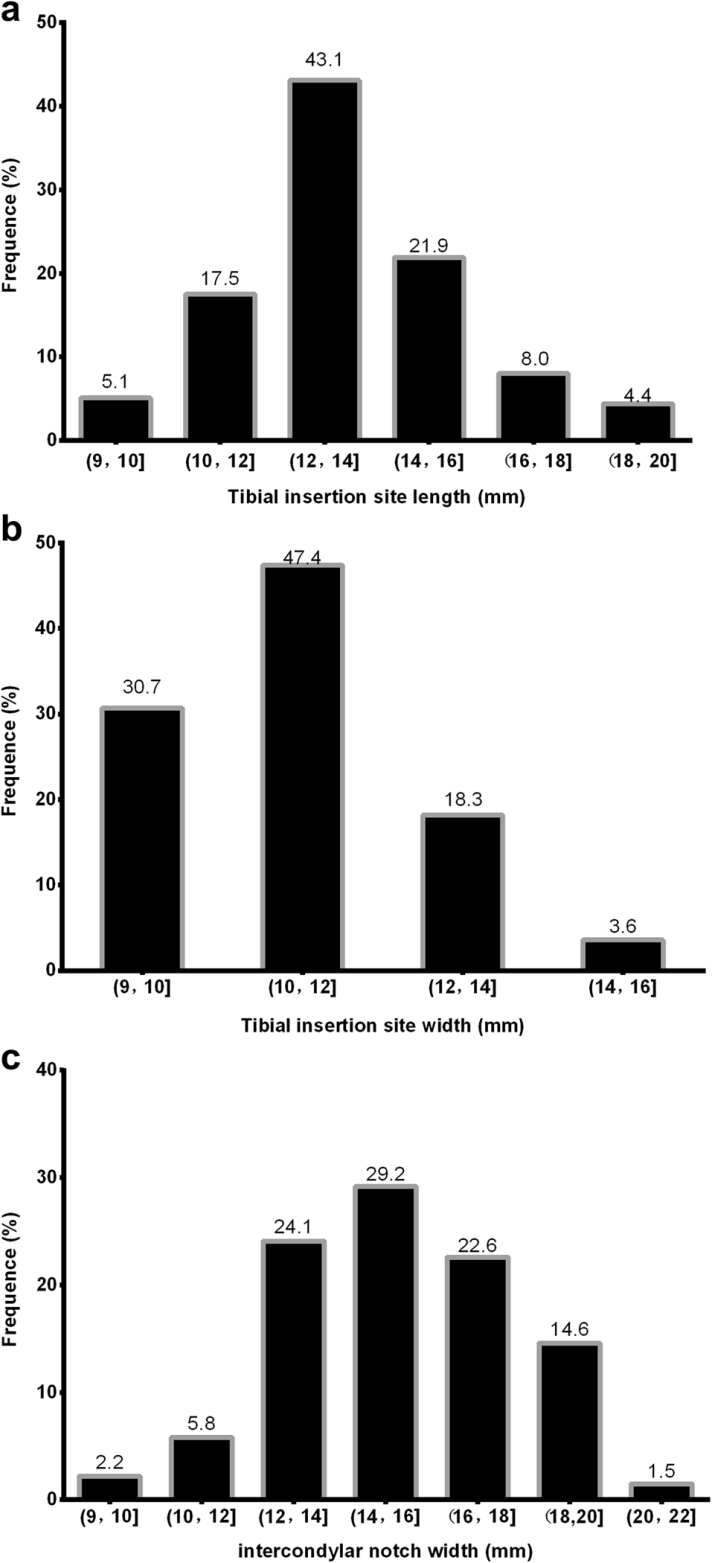
The frequency direction of the tibial insertion site length and width and the intercondylar notch width

The tibial insertion site length was variable. The distributions of the recorded lengths and widths are shown in Fig. 3a, b. On the tibial side, more than half (65.7% of the total sample) had an insertion site length that was smaller than 14 mm. The proportion of insertion sites between 14 and 16 mm was 21.9%. The proportion of insertion sites greater than 16 mm was 12.4%. The distributions of the width of the intercondylar notch are shown in Fig. 3c. The proportion of individuals with an intercondylar notch width < 14 mm was 32.1%. A total of 22.6% had an insertion site between 16 and 18 mm. A total of 16.1% had an insertion site greater than 18 mm.

## Discussion

There are two important findings in the present study. First, the sizes of the tibial insertion of the ACL and the intercondylar notch width in Chinese patients with ACL injuries were significantly different compared to those in Western patients. Second, the number of individuals whose length of ACL tibial insertion was < 14 mm accounted for 65.7% of Chinese patients with ACL injuries.

It is imperative to perform preoperative anatomical studies because the results influence the optimal selection of grafts and reconstruction techniques [29, 43]. An extensive literature review has demonstrated great variability in the tibial insertion sizes of the ACL and the intercondylar notch [8, 11, 13, 15, 16, 20, 22, 24, 36, 42]. The length of the tibial ACL insertion sites reportedly varies from 9 to 38 mm, while the insertion site width reportedly ranges from 4 to 10 mm [3, 8, 11, 21, 36]. Harald et al. [47] found a mean tibial insertion site length of 16.6 ± 1.6 mm (11.9–21.0 mm) as measured by MRI and a mean length of 16.4 ± 1.6 mm (11.0–20.0 mm) as assessed by intra-operative measurements using rulers in 146 patients who underwent primary ACL-R. Kopf et al. [22] documented that the tibial ACL insertion site had a mean length of 17.0 ± 2.0 mm in 137 patients who underwent ACL-R as measured with an arthroscopic ruler. Femke et al. [43] reported that the length of the tibial ACL insertion site was 17 ± 2.3 mm (12.0–22.0 mm) and the width was 10 ± 1.2 mm (7.0–14.0 mm) in patients who underwent arthroscopic ACL-R. However, some findings showed that the tibial footprint size in Asians is smaller than that in Western populations [17, 23, 27]. A previous study reported that the average ACL tibial attachment length was 15.36 ± 2.33 mm and the average width was 11.03 ± 1.77 mm in 77 cadaveric knees of the Thai population as assessed by direct measurement [23]. Meanwhile, another study of 100 Japanese patients with knee pain who underwent radiography and MRI showed that the average ACL tibial length was 15.2 ± 1.9 mm (14.8–15.5 mm) [17]. South Korean researchers performed ACL tibial length intra-operative measurements in 127 patients who underwent primary total knee arthroplasty, and the average tibial footprint was 13.8 mm (10.0–18.0 mm) in length and 9.8 mm (6.3–13.5 mm) in width [27].

Intercondylar notch width also has significant variability and is affected by gender. Harner et al. [14] showed that the mean notch width in patients with non-contact ACL tears was 18.4 mm. Femke et al. [48] examined patients undergoing ACL-R, and the notch width base was 16 ± 2.9 mm (10–21 mm). Similarly, Yang et al. found that the intercondylar notch width was 17.3 ± 2.1 mm in 40 Chinese patients with unilateral ACL rupture [26]. In the largest series reviewed by Shelbourne et al., the mean notch width in 714 consecutive patients who underwent ACL-R was 13.9 ± 2.2 mm for women and 15.9 ± 2.5 mm for men [33]. Thus, the conclusion was that women have smaller notches than men do [5, 9, 10, 25, 38, 40]. In another series, Shelbourne et al. and Joshua et al. also found variability between different ethnic groups, such as that African-American subjects have a larger mean intercondylar notch width than Caucasian subjects do [12, 34].

In those studies, the variation in insertion site sizes and intercondylar notch sizes may be a result of the age, gender, physique, differences in tissue quality, measuring techniques, and the race of the individuals. Iriuchishima et al. suggested that the differences in ACL size were more likely attributable to the generational differences in body size [19]. However, previous studies have failed to report exact findings in the Asian population. The available study results were limited by small sample sizes, the use of cadaveric specimens with degenerative changes, the inclusion of patients who did not receive ACL-R and measurements performed using a single technique. Therefore, these results were fundamentally flawed [18, 31, 32, 47]. To fill the knowledge gap, in addition to improving the number of research subjects and inclusion criteria, the measurements should also be carried out by different methods to obtain accurate results. Our research provides the desired data and found that the sizes of the ACL tibial insertion and intercondylar notch are relatively smaller among Chinese ACL injuries.

In the studies performed by the University of Pittsburgh, most of the ACL tibial insertion lengths among the patients undergoing ACL-R were between 16 and 18 mm [17, 22, 39, 43, 47]. Harald et al. [47] and Femke et al. [48] found lower proportions of ACL tibial insertion site length < 14 mm were 10% and 20%, respectively, as assessed by MRI and an intra-operative ruler. In a study by Kopf et al. [22] 3.6% of knees were found to have ACL tibial insertion site lengths < 14 mm. A total of 80.3% of knees found an ACL tibial insertion site length between 16 and 18 mm. Mohsen et al. [39] determined the distributions of the total ACL insertion site length and found that 19.1% of patients had lengths less than 14 mm, and 72.3% of patients had lengths between 16 and 18 mm. However, Park et al. thought that the distribution of the ACL tibial footprint size widely varied in the patient population, and they reported a higher proportion (53.5%) of tibial footprint length < 14 mm in South Korea females who underwent primary total knee arthroplasty [27]. Ichiba et al. [17] studied 100 Japanese patients with knee joint pain, and their report showed that the proportion of patients with ACL tibial footprint length < 14 mm was 28% as assessed via sagittal view on MRI. These findings showed that the proportion of tibial footprint size in Asians seems to be different from that in Western populations. However, there was no relative or exact research about the characteristics of ACL injuries among Asian populations. The present study confirms a higher proportion (65.7%) of subjects with a tibial footprint size < 14 mm in the Chinese population of patients with ACL injuries.

Fu et al. suggested that a tibial insertion site length < 14 mm and notch width < 12 mm might be better reconstructed with an SB ACL-R [22, 41]. Siebold et al. suggested an SB ACL-R may be recommended for wide insertion sites up to 16 mm in length for complete footprint restoration [35, 37]. However, a small ACL tibial footprint can also undergo DB ACL-R. Chae et al. [4] described a surgical technique of DB ACL-R with a single tibia tunnel among 22 Korean patients with small ACL tibial footprints. The research showed significantly improved results. The size of the tunnel aperture is determined by the drill-guide angle and drill bit diameter, and the DB technique also requires consideration of a single graft size [20]. For ideal, individualized, anatomical ACL-R of Chinese ACL injuries, concerns exist regarding tibial bone-tunnel orientation, drill bit diameter, and SB graft size. Much care should be taken in the Chinese population when treating patients with a DB technique or larger graft options. Based on our results, such information about reconstructed individualized ACL ruptures in Chinese patients and decisions about the choice of graft in Chinese patients undergoing ACL-R may provide usefulness to surgeons.

A limiting factor is related to the preoperative MRI in the study. The MR images were acquired after the injury. Post-injury-associated changes may obscure tibial insertion site measurements. Another limiting factor pertains to the intra-operative measurements. The length of the insertion site was measured by the midcentral portal, and the real insertion site length might not always be accurately assessed. The loss of fibres caused by the injury may result in an underestimation of the insertion site measurement. However, our senior surgeon has a great deal of experience in identifying the ACL insertion site. Finally, MRI and intra-operative measurements were obtained only by a single observer, and we were unable to evaluate the intra- and inter-observer variability. Even so, we performed the measurements both on MRI and intra-operatively and avoided measurement and distortion errors through a standardized protocol. In addition, the measurements also showed a high confidence and accuracy. Based on the above analyses, we believed the measurements were authentic.

## Conclusion

The results of the study showed the sizes of the ACL tibial insertion and intercondylar notch width in Chinese patients with ACL injuries. The data suggest that the proportion of the Chinese population with a small tibial footprint size seems different from that of the Western population. There is a higher proportion of subjects with a tibial footprint size < 14 mm in the Chinese population of patients with ACL injuries. Based on these anatomical characteristics, great care should be taken in the Chinese population when treating ACL rupture with a DB technique or larger graft options.
